# Genomic and immune microenvironment features influencing chemoimmunotherapy response in gastric cancer with peritoneal metastasis: a retrospective cohort study

**DOI:** 10.1097/JS9.0000000000001281

**Published:** 2024-03-19

**Authors:** Pengfei Yu, Guangyu Ding, Xingmao Huang, Chenxuan Wang, Jingquan Fang, Ling Huang, Zeyao Ye, Qi Xu, Xiaoying Wu, Junrong Yan, Qiuxiang Ou, Yian Du, Xiangdong Cheng

**Affiliations:** a Department of Gastric Surgery, Zhejiang Cancer Hospital, Hangzhou Institute of Medicine (HIM), Chinese Academy of Sciences; b Department of Medical Oncology, Zhejiang Cancer Hospital, Hangzhou Institute of Medicine (HIM), Chinese Academy of Sciences, Hangzhou, Zhejiang; c Medical department, Nanjing Geneseeq Technology Inc., Nanjing, Jiangsu, People’s Republic of China

**Keywords:** anti-PD1, conversion therapy, gastric cancer, peritoneal metastasis, predictive biomarker

## Abstract

**Background:**

Patients with peritoneal metastasis (PM) from gastric cancer (GC) exhibit poor prognosis. Chemoimmunotherapy offers promising clinical benefits; however, its efficacy and predictive biomarkers in a conversion therapy setting remain unclear. The authors aimed to retrospectively evaluate chemoimmunotherapy efficacy in a conversion therapy setting for GC patients with PM and establish a prediction model for assessing clinical benefits.

**Materials and methods:**

A retrospective evaluation of clinical outcomes encompassed 55 GC patients with PM who underwent chemoimmunotherapy in a conversion therapy setting. Baseline PM specimens were collected for genomic and transcriptomic profiling. Clinicopathological factors, gene signatures, and tumor immune microenvironment were evaluated to identify predictive markers and develop a prediction model.

**Results:**

Chemoimmunotherapy achieved a 41.8% objective response rate and 72.4% R0 resection rate in GC patients with PM. Patients with conversion surgery showed better overall survival (OS) than those without the surgery (median OS: not reached vs 7.82 m, *P*<0.0001). Responders to chemoimmunotherapy showed higher *ERBB2* and *ERBB3* mutation frequencies, *CTLA4* and *HLA-DQB1* expression, and CD8+ T cell infiltration, but lower *CDH1* mutation and naïve CD4+ T cell infiltration, compared to nonresponders. A prediction model was established integrating *CDH1* and *ERBB3* mutations, *HLA-DQB1* expression, and naïve CD4+ T cell infiltration (AUC=0.918), which were further tested using an independent external cohort (AUC=0.785).

**Conclusion:**

This exploratory study comprehensively evaluated clinicopathological, genomic, and immune features and developed a novel prediction model, providing a rational basis for the selection of GC patients with PM for chemoimmunotherapy-involved conversion therapy.

## Graphical abstract

**Figure:**
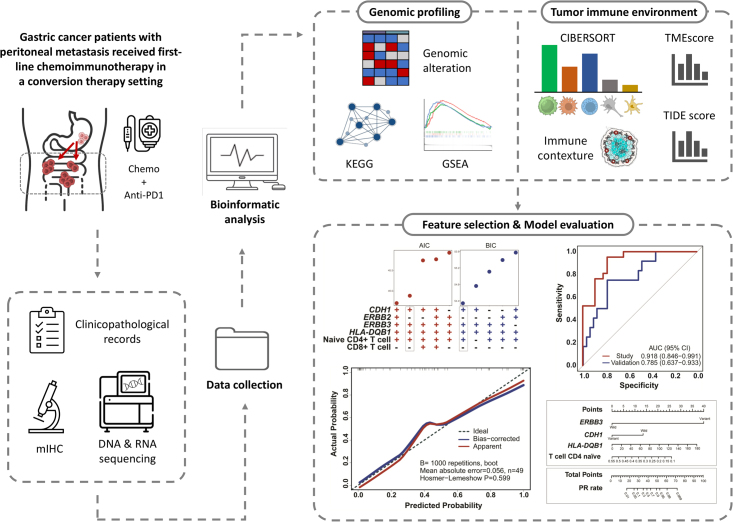

## Introduction

Highlights

- Chemoimmunotherapy achieved a 41.8% objective response rate and 72.4% R0 resection rate in gastric cancer patients with peritoneal metastasis.
- Higher *ERBB2* and *ERBB3* mutation frequencies were associated with better treatment response.
- Higher *CTLA4* and *HLA-DQB1* expression was associated with better treatment response.
- Higher infiltration of CD8+ T cells and lower infiltration of naïve CD4+ T cells were associated with better treatment response.

Gastric cancer (GC) was estimated as the fourth most prevalent cause of cancer-related death worldwide in 2020^1^. As the majority of GC cases are diagnosed in advanced stages of the disease with unsatisfactory treatment options, the 5-year survival rate is less than 10% for patients with metastatic GC^2^. Peritoneal metastasis (PM) is one of the most common metastases from GC, being detected in nearly 20% of patients upon diagnosis and in over half of the stage T3/T4 GC patients following radical resection^3^. Patients with PM of GC origin generally associate with exceedingly poor prognosis, resulting in a median survival of 4–9 months due to the aggressiveness of the disease^3^; thus, palliative chemotherapy remains the primary treatment approach for these patients in current practice. While several randomized trials have showed that systemic chemotherapy and hyperthermic intraperitoneal chemotherapy (HIPEC), combined with cytoreductive surgery (CRS), improve overall survival (OS) in patients with PM from GC^4^, limited efficacy has been observed in these patients due to inadequate tumor response to chemotherapy. Chemoimmunotherapy (i.e. immunotherapy combined with chemotherapy) has recently highlighted its significant therapeutic potential in treating various cancer types, including advanced GC^5^. Evidence suggests that immunotherapy enhances the antitumor activity of chemotherapeutic drugs by exerting a synergistic effect^5^. Promisingly, recent advances in using chemoimmunotherapy as first-line treatment significantly improved tumor response rate and/or OS in advanced GC patients compared to chemotherapy alone^6,7^.

Conversion therapy refers to the treatment approach for GC patients with initially unresectable or incurable tumors^8^. It involves chemotherapy, radiation therapy, targeted therapy, immunotherapy, or a combination of these therapies, aiming to achieve R0 resection via conversion surgery in initially unresectable tumors^8^. Recent studies suggested that advanced GC patients who received immune checkpoint inhibitor (ICI)-combined conversion therapy showed a remarkable R0 resection rate and prolonged survival. For example, the CO-STAR trial showed that the combination of sintilimab (an anti-PD-1 monoclonal antibody), chemotherapy, and apatinib treatment prior to conversion surgery showed an objective response rate (ORR) of 61.1% and a R0 resection rate of 47.2% in metastatic GC patients^9^. A retrospective study found that unresectable GC patients treated with intra-arterial chemotherapy and sequential anti-PD-1 antibody showed an ORR rate of 77.8% and an R0 resection rate of 69.4%, as well as markedly improved OS and progress-free survival^10^.

Although ICI-combined conversion therapy has shown favorable prognostic outcomes and tumor response in patients with unresectable GC, significant issues remain to be clarified. For example, little evidence supports the clinical efficacy of ICI-combined conversion therapy for individuals with GC-associated PM, which warrants investigation. Moreover, only 20–30% of patients are responders to ICI therapy^11^, highlighting a critical role of effective biomarkers for the prediction of clinical benefits and selection of appropriate patients; however, the presently developed biomarkers, such as PD-L1 expression, tumor mutational burden (TMB), and microsatellite instability (MSI), have generally showed unsatisfactory predictive power^12,13^. Therefore, this retrospective study aimed to evaluate the efficacy of systemic chemoimmunotherapy in a conversion therapy setting for GC patients with PM. The study also analyzed clinicopathological factors, gene signatures, and tumor immune microenvironment, aiming to identify biomarkers associated with treatment efficacy for the development of a prediction model.

## Material and methods

### Patients and sample collections

Our study cohort was obtained from a registered retrospective trial study. A total of 181 advanced GC patients who received first-line chemoimmunotherapy at a single-center local hospital between June 2020 and June 2022 were retrospectively evaluated. This study focused on GC patients with PM. The inclusion criteria were as follows: (1) histologically confirmed advanced primary gastric/esophago-gastric junction adenocarcinoma; (2) positive peritoneal cytology or limited peritoneal dissemination (peritoneal carcinomatosis index, PCI≤20) with or without ovarian metastasis, which was confirmed by diagnostic staging laparoscopy (DSL); (3) no prior treatment (e.g. radiotherapy, chemotherapy, targeted therapy, or immunotherapy); (4) age of 18–75 years; (5) Eastern Cooperative Oncology Group performance status (PS) 0–1^14^; and (6) adequate organ function [alanine transaminase and aspartate transaminase levels < twice the upper limit of normal (ULN); serum total bilirubin <1.5 times the ULN; serum creatinine <1.25 times the ULN; platelet count ≥100×10^9^/l; absolute granulocyte counts ≥1.5×10^9^/l; and hemoglobin level ≥90 g/l]. The exclusion criteria were as follows: (1) concomitant malignancies or refractory autoimmune diseases; (2) a long-term history of steroid or immunosuppressant use; (3) diffuse PM (PCI>20) or with other distant metastases; (4) lack of complete clinicopathological data.

**Figure 1:**
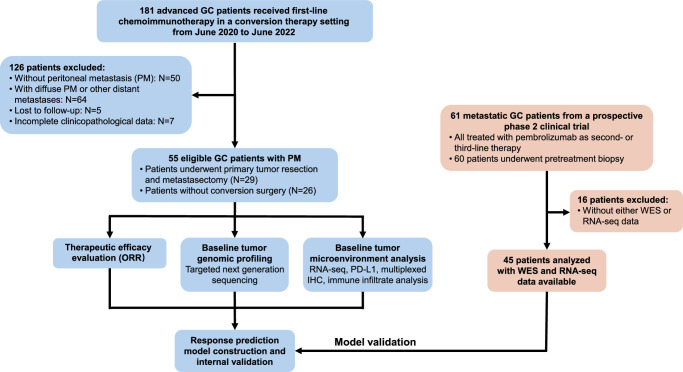
Schematic demonstration of the study design. A total of 55 eligible gastric cancer (GC) patients with peritoneal metastasis (PM) were included in this study. Targeted next generation sequencing (NGS) and RNA sequencing (RNA-seq) on PM specimens were performed. Tumor immune microenvironment was analyzed. A model for predicting treatment response was constructed and validated using an external validation cohort.

According to the above criteria, a total of 55 patients were included in the subsequent analyses. Baseline peritoneal tumor specimens fixed in a formalin solution and embedded in paraffin were retrospectively collected for genomic profiling and tumor microenvironment (TME) analyses. The flowchart of patient enrollment and study design is presented in Figure 1. All patients provided informed consent to participate. This study has been reported in line with the strengthening the reporting of cohort, cross-sectional, and case–control studies in surgery (STROCSS) criteria^15^ (Supplemental Digital Content 1, http://links.lww.com/JS9/C111).

### Conversion therapy

All patients underwent imaging examinations and DSL to assess the extent of disease and resectability before initiating treatment. The first-line chemoimmunotherapy regimen involved sintilimab in combination with chemotherapy based on body surface area (BSA). Within a 3-week cycle, sintilimab (200 mg, q3w) and paclitaxel (150 mg/m^2^) were intravenously administered on day 1, and S-1 (BSA <1.25 m^2^, 40 mg/d; 1.25 ≤BSA <1.50 m^2^, 50 mg/d; BSA ≥1.50 m^2^, 60 mg/d) was administered on days 1–14. Chemoimmunotherapy was administered for 2–6 cycles, and the decision to perform surgical resection was made by consensus from multidisciplinary team assessments every two cycles.

Patients were considered eligible for conversion surgery following the completion of the last chemoimmunotherapy cycle: (1) disappearance or remarkable shrinkage of PM by imaging examinations, (2) no other distant metastases or unresectable metastases, (3) downstaging of the primary tumor, (4) clinical symptoms dramatically resolved. CRS was undertaken when intraoperative exploration indicated complete resectability of the primary tumor and metastases (Supplementary Figure 1, Supplemental Digital Content 2, http://links.lww.com/JS9/C112). If radical resection was not feasible, a peritoneal tumor biopsy was conducted for further assessment. HIPEC was performed twice within 72 h after each DSL or CRS. Patients who underwent conversion surgery continued their original chemoimmunotherapy regimens for 4–6 cycles after the surgery, followed by S1 combined with sintilimab for up to 1 year. For those not meeting eligibility criteria for, or expressing hesitancy about, conversion surgery, their original chemoimmunotherapy regimen was continued for 4–6 cycles, followed by S-1 and sintilimab for up to 2 years or until disease progression.

### Clinical assessments

Tumor response was assessed according to the Response Evaluation Criteria in Solid Tumors (RECIST) guidelines version 1.1 based on imaging examinations and maximum tumor diameter change^16^. The radiological response was graded into complete response (CR), partial response (PR), stable disease (SD), and progressive disease (PD)^16^. Surgical classification was categorized as gross residual disease (R2), positive margin of resection (R1), or complete resection with negative margins (R0)^17^. Histological tumor regression grade (TRG) was used to assess tumor regression in surgical specimens. TRG was defined as follows: TRG 1a, no residual tumor cells; TRG 1b, <10% residual tumor cells; TRG 2, 10–50% residual tumor cells; TRG 3, >50% residual tumor cells^18^.

Follow-up was performed once every 3 months for the first 2 years and once every 6 months thereafter. The follow-up methods mainly included telephonic follow-ups and regular outpatient reexaminations. OS was defined as the time interval from the date of pathological diagnosis of GC to the date of death or the most recent follow-up. Patients who did not die at the last follow-up were censored. The cutoff date for OS is 31 January 2023.

### DNA extraction and library preparation

DNA extraction, quantification, and library preparation were performed as previously described^19^. Detailed descriptions were provided in Supplementary Methods (Supplemental Digital Content 3, http://links.lww.com/JS9/C113).

### Targeted next generation sequencing (NGS) and data processing

The DNA libraries were paired-end sequenced using Illumina HiSeq4000 NGS platforms (Illumina) as previously described^19^. Detailed descriptions were provided in Supplementary Methods (Supplemental Digital Content 3, http://links.lww.com/JS9/C113). The cancer-related pathways were selected according to a previous study^20^ and KEGG database. The DNA damage response (DDR) pathways were selected as per previously reported^21^. The immune-related pathways were selected according to ImmPort database.

### RNA sequencing (RNA-seq)

RNA extraction and sequencing were performed as previously described^22^. Detailed descriptions were provided in Supplementary Methods (Supplemental Digital Content 3, http://links.lww.com/JS9/C113).

### Gene expression and pathway analysis

Basecalling was performed on Illumina bcl2Fastq software (v2.19.0.316) to generate sequence reads in FASTQ format. Sequences were trimmed using Trimmomatic software (v0.36) prior to assembly^23^. Transcriptomic mapping was performed using STAR software (v2.7.3a) and aligned to the reference human genome (hg19). RSEM (v1.2.31) was used for gene level quantification. Differential expression analysis was performed using the DESeq2 R package based on negative binomial distribution. The gene expression differences between the two groups were considered significant if |log_2_FC|≥2 and FDR-adjusted *P*-value (P.adjust) ≤0.1. Gene set enrichment analysis (GSEA), Gene Ontology (GO) term enrichment, and Kyoto Encyclopedia of Genes and Genomes (KEGG) pathway analysis were conducted using the clusterProfiler R package (v4.4.4) with a cutoff of P.adjust<0.05.

### Immune infiltrate analysis

Immune infiltrating cells were assessed and quantified using CIBERSORT^24^. TMEscore (TME score) was estimated using principal component analysis based on RNA-seq data. Genes associated with different TME phenotypes were analyzed using the R package TMEscore (https://github.com/DongqiangZeng0808/TMEscore). TMEscore A was associated with immune-related signatures, while TMEscore B was associated with stromal-relevant signatures^25^. T cell dysfunction and exclusion scores was evaluated using Tumor Immune Dysfunction and Exclusion (TIDE, http://tide.dfci.harvard.edu)^26^.

### Immunohistochemistry (IHC)

PD-L1 expression was determined using a Dako PD-L1 IHC 22C3 pharmDx kit (Agilent Technologies) in combination with the Dako Autostainer Link 48 system (Agilent Technologies). The PD-L1 expression level was evaluated by combined positive score (CPS), and CPS≥1 was identified as positive^27^.

### Multiplexed IHC (mIHC) and multispectral imaging

Multiplex immunofluorescence staining was performed using a PANO 7-plex IHC kit (Panovue) as per manufacturer’s instructions. Detailed descriptions were provided in Supplementary Methods (Supplemental Digital Content 3, http://links.lww.com/JS9/C113).

### Prediction model construction and validation

Complete subset regressions for all combinations of candidate features were used to select the best fitting model (R package, leaps). Akaike information criteria (AIC) and Bayesian information criteria (BIC) were used to measure candidate model performance and select features for subsequent prediction model construction. A prediction model that consisted of the selected features was built by logistic regression and presented as a nomogram using the rms R package. The predictive performance was evaluated based on the area under the ROC curve (AUC), accuracy, sensitivity, and specificity (R package, pROC). The calibration of the nomogram was assessed by a Hosmer–Lemeshow (HL) test^28^. Internal validation was performed using the bootstrapping method (1000 bootstrap resamples). The whole exome sequencing (WES) and RNA-seq data of an independent cohort was obtained from a previous study^29^ and used for external validation (Fig. 1). In brief, this external cohort involved a total of 61 metastatic GC patients from a prospective phase II clinical trial. All of them were Korean, with a median age of 57 years (range, 26–78 years), and the majority were male (70.5%). They received pembrolizumab as a second-line or third-line treatment. Most biopsy samples were obtained from stomach/primary tumor (46/61), while other samples were obtained from metastatic sites. Of the 61 patients, 16 patients were excluded from our study analysis due to the lack of WES and/or RNA-seq data. All analyses were conducted using R software (v4.1.2).

### Statistical analysis

All statistical analyses were performed using R software (v4.1.2). A Wilcoxon rank sum test, *t*-test, or Fisher’s exact test was used to compare differences between groups. OS curves were generated using the Kaplan–Meier method and compared using a log-rank test. A two‐tailed *P*-value ≤0.05 was considered statistically significant unless indicated otherwise.

## Results

### Patient characteristics

A total of 55 unresectable GC patients with PM who underwent ICI-combined conversion therapy were retrospectively evaluated. The patients (25 males and 30 females) had a median age of 55 years, ranging from 26 to 75 years. Gastric tumors were mostly located at corpus (54.4%) or antrum (40.0%). Histological assessment showed that 80.0% of tumors were poorly differentiated. According to Lauren’s classification, 31 patients had diffused GC, while others had either intestinal (14/55) or mixed (10/55) GC. Based on imaging assessment, multidisciplinary team consultation, and personal preference, 29 out of 55 patients proceeded with conversion surgery (i.e. primary tumor resection and metastasectomy), resulting in a conversion rate of 52.7%. Of the 29 patients, 21 (72.4%) achieved R0 resection. Other clinicopathological characteristics of all patients are summarized in Supplementary Table 1 (Supplemental Digital Content 4, http://links.lww.com/JS9/C114). Grades 3 or 4 adverse events were identified in 16 of the 55 patients (29.1%) in the chemoimmunotherapy stage (Supplementary Table 2, Supplemental Digital Content 4, http://links.lww.com/JS9/C114). All complications were adequately controlled by conservative treatment.

### Conversion surgery improves prognosis in unresectable GC patients with PM

**Figure 2:**
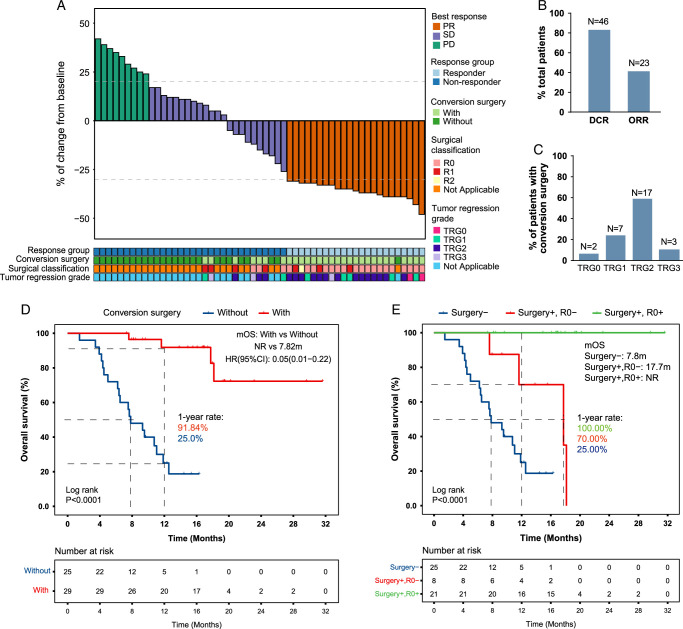
Therapeutic response to chemoimmunotherapy and overall survival of gastric cancer (GC) patients with peritoneal metastasis (PM). (A) Waterfall plot showing the summary of patient response to chemoimmunotherapy within the context of conversion therapy. (B) Disease control rate (DDR) and overall response rate (ORR) in patients. (C) Tumor regression grade (TRG) in patients who underwent conversion surgery. (D) Kaplan–Meier curve of overall survival in patients with or without conversion surgery. (E) Kaplan–Meier curve of overall survival in patients without conversion surgery (Surgery-), with conversion surgery but did not achieve R0 resection (Surgery+, R0-), or with conversion surgery and R0 resection (Surgery+, R0+). A log-rank test was used to compare overall survival between groups. A *P*-value≤0.05 was considered statistically significant. NR, not reached.

The median follow-up time of all patients was 11.3 months (range: 2–32 months). Of the 55 patients who had conversion therapy, the numbers of patients who achieved PR, SD, and PD were 23, 23, and 9, respectively (Fig. 2A), resulting in a disease control rate of 83.6% and an ORR of 41.8% (Fig. 2B). In this study, patients who achieved PR were categorized into responders, while those with SD/PD were grouped as nonresponders. The numbers of patients who achieved TRG 1a, TRG 1b, TRG 2, and TRG 3 were 2, 7, 17, and 3, respectively (Fig. 2C). As expected, patients with conversion surgery had significantly higher OS than those without the surgery (mOS: not reached vs 7.82 months, *P*<0.0001; Fig. 2D). Moreover, the R0 resection group had significantly higher OS than the R1 or R2 resection group (mOS: not reached vs 17.7 months, *P*<0.001; Fig. 2E).

### Clinicopathological and genetic features predict patient response to ICI-combined conversion therapy

**Figure 3:**
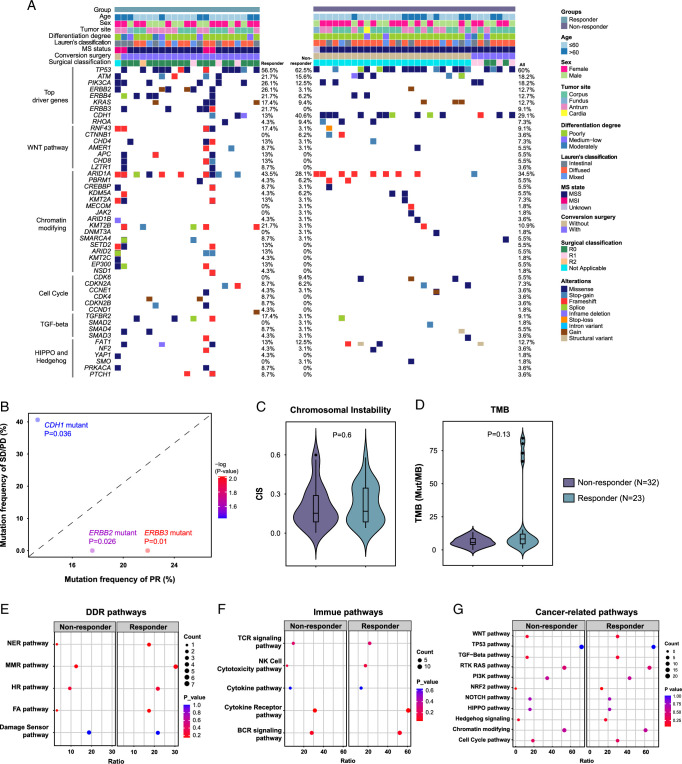
Baseline genetic features of peritoneal metastasis (PM) specimens from gastric cancer (GC) patients. (A) Somatic genomic alterations identified in PM specimens using targeted next generation sequencing (NGS). The variant frequencies were calculated in all patients, as well as in responders and nonresponders. (B) Comparison of gene variant frequencies between nonresponders and responders. A Fisher’s exact test was used to compare variant frequencies between groups. (C, D) Chromosomal instability score (CIS) and tumor mutational burden (TMB) were compared between nonresponders and responders using a Wilcoxon rank sum test. (E–G) DNA damage response (DDR), immune-related, and cancer-related pathway analyses for mutations enriched in nonresponders vs. responders.

To identify predictive markers for conversion therapy in our study, we compared responders (*N*=23) and nonresponders (*N*=32) based on their clinicopathological, genetic, and immune features. A significantly higher number of responders were found with CA125≤35 U/ml or MSI-H compared to nonresponders (*P*<0.001 and *P*=0.028, respectively), whereas no differences were found in other measured clinical characteristics between the two groups (Table 1). Notably, no differences were found in PD-L1 CPS between the two groups, indicating the importance of identifying effective biomarkers for prediction of ICI-combined conversion therapy. Targeted NGS identified genetic variations in these patients, with the top five frequently mutated genes being *TP53, ARID1A, CDH1, LRP1B*, and *ATM* (Fig. 3A). The identified variant genes mostly belong to cancer driver genes, WNT pathways, chromatin modification, and cell cycle (Fig. 3A). Regrettably, the exploration of genes associated with treatment response yielded no individual genes meeting an FDR≤0.05, which may be partially attributed to the limited sample size and the overall low mutation frequency. However, variant frequencies of three cancer driver genes tended to be distinct between responders and nonresponders. The responders showed higher frequencies in *ERBB3* (21.7 vs 0%, *P*=0.010) and *ERBB2* (17.4 vs 0%, *P*=0.026) variations but a lower frequency in *CDH1* variations (13.0 vs 40.6%, *P*=0.036), compared to nonresponders (Fig. 3B). Other identified genetic variations showed no differences between the two groups. In addition, no differences were found in CIS and TMB between responders and nonresponders (Fig. 3C, D). Furthermore, no differences were found in DDR, immune-related, or cancer-related pathways between the two groups (Fig. 3E–G).

**Table 1:** Comparisons of patient characteristics between nonresponders and responders.

Characteristics	Nonresponder (*N*=32)	Responder (*N*=23)	*P*
Age of diagnosis			1
≤60	22	15	
>60	10	8	
Sex			1
Female	17	13	
Male	15	10	
Smoking history			1
With	5	4	
Without	27	19	
Drinking history			0.435
With	3	4	
Without	29	19	
Tumor site			0.739
Antrum	11	11	
Cardia	1	0	
Corpus	19	11	
Fundus	1	1	
Histological grade			0.585
Poorly differentiated	27	17	
Medium-low differentiated	4	4	
Moderately differentiated	1	2	
Lauren’s classification			0.597
Diffused	20	11	
Intestinal	7	7	
Mixed	5	5	
CA199 (U/ml)			0.377
≤37	21	18	
>37	11	5	
CEA (ng/ml)			0.166
≤5	24	21	
>5	8	2	
CA125 (U/ml)			<0.001^*^
≤35	5	15	
>35	27	8	
*ERBB2* amplification (FISH/IHC)			1
No	20	19	
Yes	1	1	
Unknown	11	3	
MS status (NGS)			0.028^*^
MSS/MSI-L	31	19	
MSI-H	0	4	
Unknown	1	0	
PD-L1 (22C3, CPS)			0.243
<1	15	6	
≥1	15	14	
Unknown	2	3	
Cycle number of chemoimmunotherapy			
≤4	18	13	1
>4	14	10	

Data was analyzed using a Fisher’s exact test.

* Indicates statistical significance.

CA, cancer antigen; CEA, carcinoembryonic antigen; CPS, combined positive score; MSI-H, high microsatellite instability; MSS/MSI-L, Microsatellite stable/low microsatellite instability.

### Immune-related features predict patient response to ICI-combined conversion therapy

**Figure 4:**
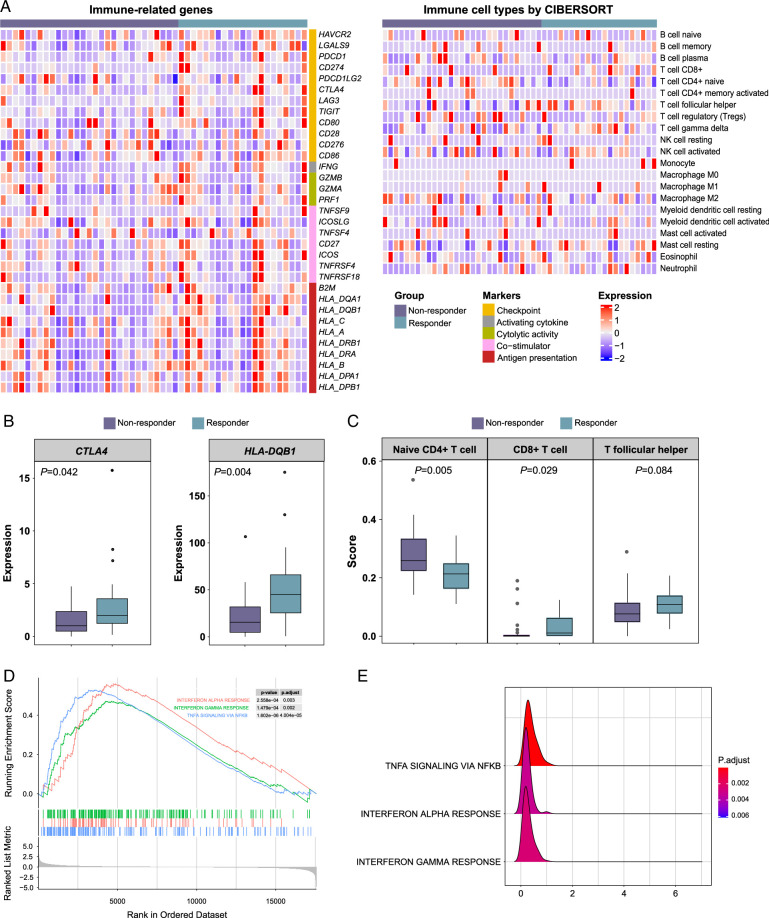
Immune-related features of peritoneal metastasis (PM) specimens from gastric cancer (GC) patients. (A) Immune-related gene expression profiling using RNA-seq data, and relative fraction of 21 immune cell types inferred by CIBERSORT. (B) Comparison of CTLA4 and HLA-DQB1 expression between nonresponders and responders using a Wilcoxon rank sum test. (C) Comparison of naïve CD4+ T cell, CD8+ T, and T follicular helper cell infiltration between nonresponders and responders using a Wilcoxon rank sum test. (D, E) GSEA pathway analysis using hallmark gene sets. The gene set under the pathway of |NES|>1 and FDR-adjusted *P*<0.05 was considered significant between nonresponders and responders. NES, normalized enrichment score; FDR, false discovery rate.

RNA-seq analyses identified nine upregulated and two downregulated genes (Supplementary Figure 2A, Supplemental Digital Content 2, http://links.lww.com/JS9/C112). As immunotherapy was a major part of our conversion therapy, we next focused on analyzing tumor immune-related gene expression and immune cell types using RNA-seq data (Fig. 4A). *CTLA4* and *HLA-DQB1* expression was higher in responders than that in nonresponders (Fig. 4B), whereas other immune-related genes showed no differences between the two groups. A lower level of naïve CD4+ T cell infiltration (*P*=0.0050) and a higher level of CD8+ T cell infiltration (*P*=0.029) were found in responders compared to nonresponders (Fig. 4C), indicating their potential in predicting a better treatment response. The responders also had a higher trend of T follicular helper cell (Tfh) infiltration but did not reach statistical significance (*P*=0.084; Fig. 4C). GSEA analysis showed that responders had an upregulation of immune-related pathways, including interferon gamma-response (IFNγ), interferon-alpha response (IFNα), and NFkB-mediated TNFα signaling pathways (Fig. 4D, E). Results from TMEscore, TIDE, and tertiary lymphoid structure (TLS) analyses did not show significant differences between the two groups (Supplementary Figure 2B, 2C&2D, Supplemental Digital Content 2, http://links.lww.com/JS9/C112).

### *ERBB3* mutations may serve as a strong biomarker to predict patient response to ICI-combined conversion therapy

**Figure 5:**
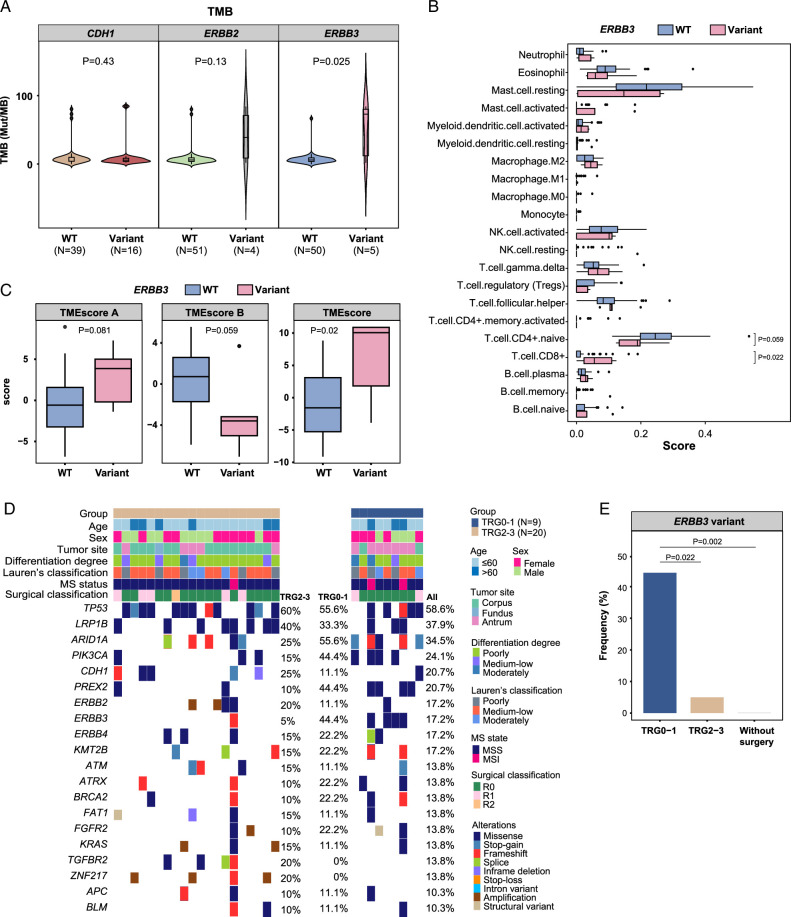
*ERBB3* mutations as a strong biomarker to predict treatment response in gastric cancer (GC) patients with peritoneal metastasis (PM). (A) Patients with *ERBB3* variants showed a higher tumor mutational burden (TMB) than those with wildtype (WT) *ERBB3*. (B) Patients with *ERBB3* variants showed lower naïve CD4+ T cell infiltration but higher CD8+ T cell infiltration than those with WT *ERBB3*. (C) Comparison of TMEscore A, TMEscore B, and TMEscore between *ERBB3* variants and WT. (D) Somatic genomic alterations identified in PM specimens of patient underwent conversion surgery (*N*=29). The variant frequencies were calculated in all patients, as well as in the TRG0–1 and TRG2–3 group. (E) Comparison of *ERBB3* variant frequency between the TRG0–1 group, TRG2–3 group, and patients without conversion surgery. A Fisher’s exact test was used to compare variant frequencies between groups. A Wilcoxon rank sum test was used in A–C. A *P*-value ≤0.05 was considered statistically significant.

Of the three genetic variations identified (i.e. *ERBB2, ERBB3*, and *CDH1*), we found that patients with *ERBB3* mutations had a significantly higher TMB than patients with wildtype (WT) *ERBB3* (*P*=0.025), whereas *CDH1* and *ERBB2* variations were not associated with TMB (Fig. 5A). Moreover, patients with variant *ERBB3* had a significantly higher level of CD8+ T cells (*P*=0.022) and a lower trend in the activated memory CD4+ T cell level (*P*=0.059) (Fig. 5B). Additionally, a significantly higher TMEscore (*P*=0.020) was found in patients with variant *ERBB3* compared to patients with WT *ERBB3*. A higher trend in TMEscore A (*P*=0.081) and lower trend in TMEscore B (*P*=0.059) were also found in the *ERBB3* variant group (Fig. 5C). No differences were found in TIDE and TLS (Supplementary Figure 3A & 3B, Supplemental Digital Content 2, http://links.lww.com/JS9/C112) between *ERBB3* WT and variant group. Compared with patients with *ERBB3* variants, patients with *CDH1* or *ERBB2* variants had fewer differences in immune-related signatures compared to their respective WT (Supplementary Figure 3C–J, Supplemental Digital Content 2, http://links.lww.com/JS9/C112), except a higher level of naïve CD4+ T cells in *CDH1* variant group (Supplementary Figure 3D, Supplemental Digital Content 2, http://links.lww.com/JS9/C112) and higher plasma B cells in *ERBB2* variant group (Supplementary Figure 3H, Supplemental Digital Content 2, http://links.lww.com/JS9/C112).

Furthermore, we found that the *ERBB3* variant frequency was higher in the TRG1a-1b group than that in the TRG2-3 group (44.4 vs 5.0%, *P*=0.022) among patients who underwent conversion surgery, whereas frequencies of other identified genetic variations showed no significant differences between the two groups (Fig. 5D, E). Additionally, no differences were found between the two groups in TMB, CIS, expression of immune-related genes, TLS, or immune-related scores (Supplementary Figure 4A–E, Supplemental Digital Content 2, http://links.lww.com/JS9/C112).

### Prediction model for patient response to conversion therapy

**Figure 6:**
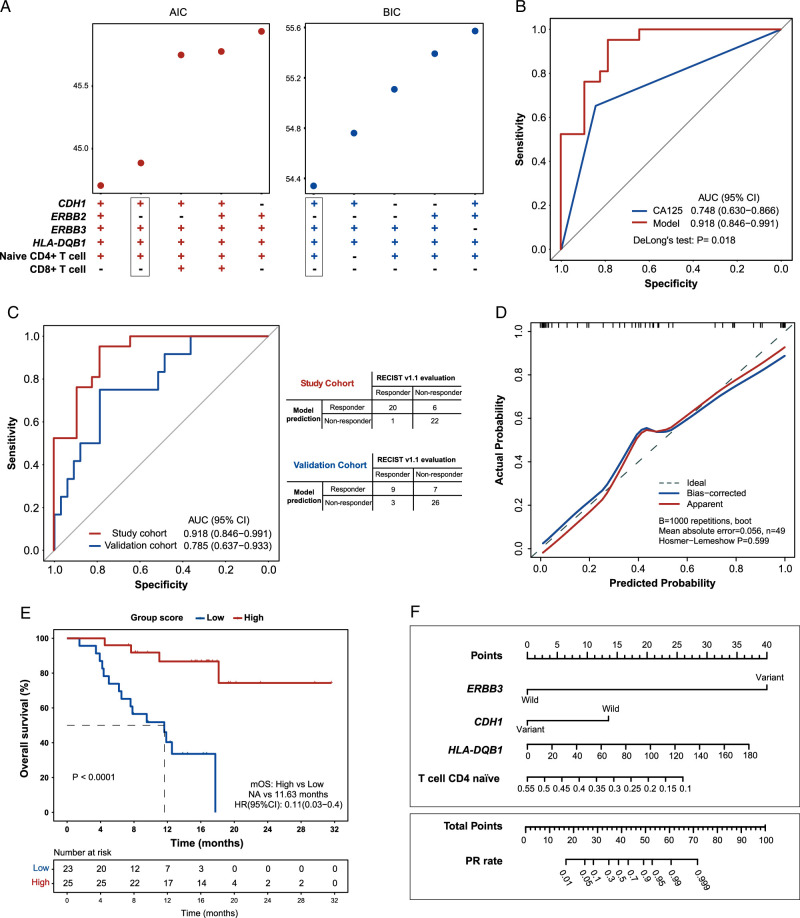
Feature selection and validation of the prediction model. (A) Optimal feature selection based on the Akaike information criteria (AIC) and Bayesian information criteria (BIC) methods. (B) Receiver operating characteristic (ROC) curves for CA125 and the model of study cohort. DeLong’s test was used to compare the difference between areas under the curve (AUC). (C) ROC curves and confusion matrices for the study cohort (*N*=55) and external validation cohort (*N*=45). (D) The prediction model was internally validated using bootstrap resampling (1000 times). The Hosmer–Lemeshow test indicated a good fit (*P*=0.599). (E) The study cohort were divided into low-score and high-score groups based on the optimal classification threshold (−0.457). The Kaplan–Meier curve showed that the high-score group had a significantly better overall survival than the low-score group (F) A nomogram was built for clinical estimation of the PR probability.

To establish a model for predicting patient response, we explored three feature selection methods: AIC and BIC, LASSO and XGboost. Considering the comparison results of the model performance (Supplementary Figure 5, Supplemental Digital Content 2, http://links.lww.com/JS9/C112) and limitations posed by our small sample size, AIC and BIC were chosen to select optimal features for model construction. As we identified eight molecular features that showed significant differences between responders and nonresponders (i.e. MSI, *CDH1, ERBB2*, and *ERBB3* variants, *CTLA4* and *HLA-DQB1* expression, CD4+ naïve, and CD8+ T cell infiltration), all combinations of these factors were analyzed in AIC and BIC models to select optimal features, and the combination of *CDH1* variant, *ERBB3* variant, *HLA-DQB1* expression, and naïve CD4+ cell infiltration was eventually selected for subsequent prediction model construction (Fig. 6A). The score of individual PR rate was calculated as follows: score=1.375 + 8.3795 × *ERBB3* + (−2.8391) × *CDH1*+ 0.043 × *HLA-DQB1* + (−12.0883) × T cell CD4 naïve. A higher score value indicates a higher probability of PR. The optimal classification threshold was determined using Youden index, yielding a cutoff value of −0.475. The final prediction model yielded an AUC of 0.918 (95% CI: 0.846–0.991), an accuracy of 0.857 (95% CI: 0.728–0.941), a sensitivity of 0.952 (95% CI: 0.762–0.999), and a specificity of 0.786 (95% CI: 0.591–0.917). Notably, our model showed significantly higher AUC than the clinical factor CA125 (0.918 vs 0.748, *P*=0.018, Fig. 6B), indicating a superior predictive performance of the presented biomarkers in contract to the simple clinical variable. The model persisted in high performance regardless of their clinical characteristics, such as PD-L1 status, Lauren’s classification, age, sex, and tumor location (Supplementary Figure 6A–F, Supplemental Digital Content 2, http://links.lww.com/JS9/C112).

We further evaluated our prediction model using the dataset obtained from an independent cohort of 45 metastatic GC patients who underwent immunotherapy as second-line or third-line treatment^29^. The validation cohort achieved an AUC of 0.785 (95% CI: 0.637–0.933), an accuracy of 0.778 (95% CI: 0.629–0.888), a sensitivity of 0.750 (95% CI: 0.428–0.945), and a specificity of 0.787 (95% CI: 0.611–0.910) (Fig. 6C). Internal validation of the model was performed using the bootstrapping method and yielded a *P*-value of 0.599 from the Hosmer–Lemeshow test, indicating a good fit of the model (Fig. 6D). Additionally, patients from this study were divided into low-score and high-score groups based on the optimal classification threshold (−0.457) as defined above. The KM analysis showed that the high-score group had a significantly better OS than the low-score group (*P*<0.0001; Fig. 6E). A nomogram was generated based on the logistic regression models for individual predictions of patient response to conversion therapy (Fig. 6F).

## Discussion

This study identified predictive biomarkers and established a novel prediction model aimed at anticipating the response to chemoimmunotherapy in GC patients with PM using comprehensive genomic and transcriptomic profiling. To the best of our knowledge, this was the first study focused on predictive biomarker investigation for the selection of GC patients with PM in a conversion therapy setting. First, we showed the efficacy of ICI-combined conversion therapy in GC patients with PM. Notably, we introduced an innovative model that integrates genetic and immune features to anticipate patient responses to chemoimmunotherapy, and the model was further evaluated using an independent external cohort. Collectively, our study provided evidence and a rational basis for the selection of GC patients with PM for ICI-combined conversion therapy. While we mostly focused on the endpoints related to the treatment response, we acknowledge that the short and diverse follow-up period may limit the broader implications of our findings. Future studies with extended follow-up durations could provide more insights into the long-term outcomes associated with the biomarkers identified in our study.

With a major focus on PM patients, our study showed that ORR (41.8%) and R0 resection rate (72.4%) of conversion therapy were comparable but tended to be lower compared to previous studies focusing on GC patients with various metastatic sites. For example, an R0 rate of 84.8% was found in 100 unresectable GC patients who underwent conversion therapy^30^. A recent study combining anti-PD1 antibody to conversion therapy showed an ORR of 77.8% and an R0 resection rate of 83.3% in 36 unresectable GC patients^10^. Additionally, a CO-STAR trial with the combination of chemotherapy, PD-1 inhibitor, and tyrosine kinase inhibitor regimens showed an ORR rate of 61.1% and a R0 resection rate of 47.2% in stage IV metastatic GC patients^9^. The relatively lower ORR and R0 resection rate observed in our study may be attributed to our targeted cohort being peritoneal metastatic patients, whereas previous studies involved GC patients with various metastatic sites. PM is more resistant to chemotherapy compared to other common metastases from GC due to low drug delivery into the abdominal cavity^31,32^, partially contributing to relatively lower efficacy in this study. Despite this difference, strong consistency was observed between our study and others regarding the significant improvement of OS in patients with conversion surgery and R0 resection^10,30,32,33^.

In the current practice, clinicopathological factors (e.g. tumor histopathology and metastasis) remain the cornerstone for predictive and prognostic evaluation in cancer treatment. Genetic and immune biomarkers have also been included, especially for identifying responders of immunotherapy, but they generally show a low predictive power. For example, several studies found no correlation between PD-L1 expression and clinical benefits of ICI therapy in advanced cancer patients^34,35^. Although compelling data suggested a significant correlation between high TMB and clinical benefits of ICI therapy^36^, increasing evidence showed that TMB alone was not capable of distinguishing responders of immunotherapy or predicting OS^37^. Similarly, our study showed that TMB and PD-L1 level failed to serve as predictive biomarkers, highlighting the necessity to explore effective biomarkers. Additionally, individual predictive factors lack accurate reflection of the complex TME and dynamically elevated tumor heterogeneity. Several studies have incorporated different signatures into the nomogram and showed higher accuracy in predicting treatment response in cancer patients^38,39^. Therefore, we performed genomic and transcriptomic profiling and established a novel prediction model that incorporated both genetic and immune-related features. The observed difference in AUC between our model and CA125 further reinforced the added value of our selected biomarkers in refining the predictive accuracy for chemoimmunotherapy response, as opposed to relying solely on basic clinical variables, suggesting the significance of genetic biomarker exploration in this field. Regrettably, the assembly of an independent validation cohort was not feasible due to inherent limitations posed by the limited cohort size and the less commonly adopted treatment approaches. Fortunately, we found a previously published study involving metastatic GC patients who underwent immunotherapy as second-line or third-line treatment^29^, with accessible raw dataset of ORR, WES, and RNA-seq. However, the validation cohort showed a decrease in AUC from 0.91 to 0.78 compared to our study cohort, which may be partially attributed to the differences in study settings, such as tumor sample origin (PM from GC vs primary GC) and the regimen of immunotherapy (first-line vs non-first-line).

*CDH1* is a tumor-repressor gene that encodes E-cadherin, and its mutations are well-recognized as risk factors for GC. A previous study identified *CDH1* mutations as independent predictive factors of ICI-resistance in patients with MSI-H gastrointestinal cancer^40^. Similarly, a phase II trial found that advanced GC patients carrying *CDH1* mutations were nonresponders of anti-PD-1 therapy^41^. Consistent with these findings, our study found that patients carrying *CDH1* mutations had a worse response to ICI-combined conversion therapy. *ERBB2* and *ERBB3* encode HER2 and HER3 proteins, respectively. *ERBB2*/*ERBB3* mutations are significant in a variety of cancers due to their ability to activate the downstream PI3K/AKT and ERK pathways in tumors, promoting cancer cell proliferation; therefore, they are commonly considered poor prognostic markers^42^. However, studies found that cancer patients with *ERBB* mutations had better response to chemoimmunotherapy than those with WT *ERBB* ^43,44^. Using mouse and cell culture models, a study found that gallbladder tumors or cancer cells harboring *ERBB2/ERBB3* mutations showed an enhanced effect of anti-PD-1 treatment compared to WT^43^. These findings suggest that *ERBB2*/*ERBB3* mutations are potential predictive biomarkers to identify appropriate patients for ICI therapy. Our study provides clinical support that *ERBB2*/*ERBB3* mutations may predict response to ICI-combined conversion therapy in GC patients with PM. Evidence also suggests that *ERBB2*/*ERBB3* mutations associate with higher PD-L1 expression^43,45^, which may explain their ability to predict clinical benefits of anti-PD-1 therapy; however, the molecular mechanisms need to be explored. Moreover, our study showed that patients with *ERBB3* mutations had higher CD8+ T cell infiltration, TMEscore, and TMB, indicating a potentially better response to immunotherapy; however, these immune signature differences were not observed in patients with *ERBB2* or *CDH1* mutations. Therefore, *ERBB3* mutations may serve as robust predictive biomarkers of ICI-combined conversion therapy in GC patients with PM.

Tumor immunity is essential in therapeutic response, especially in immunotherapy-involved regimens^46^. As anti-PD-1 antibody treatment was an essential part of conversion therapy in our study, we comprehensively evaluated immune response-related gene expression, specific tumor-infiltrating immune cells, and immune-related scores using transcriptomic data. *HLA* is a group of highly polymorphic gene complex that activates host immune response^47^. Of all the tested *HLA* subtypes, only *HLA-DQB1* expression significantly correlated with therapeutic response in our study. Although studies reported *HLA-DQB1* being a promising prognostic marker in cancer patients^48,49^, limited evidence was found regarding its predictive value of ICI therapy in GC patients, which warrants further validation. Emerging evidence suggests that infiltration of immune cells in TME holds both predictive and prognostic value in immunotherapy. For example, studies suggested that higher density and expression of CD8+ T cells in tumor was directly associated with better response to anti-PD-1 treatment in a variety of cancers^50^. Similarly, we found that GC patients with higher CD8+ T cell infiltration had better therapeutic response. A higher level of naïve CD4+ T cells indicates worse adaptive immunity^51^, which aligns with our findings showing a worse treatment response in patients with a higher infiltration level of naïve CD4+ T cells. However, limited clinical evidence was found regarding the predictive value of naïve CD4+ T cell infiltration for immunotherapy in cancer patients, which needs to be further explored in future studies.

TME is a complex and dynamic network that contains diverse infiltrating and resident cells, blood vessels, extracellular matrix, and signaling molecules; therefore, several scoring systems have been developed to quantitatively characterize the overall immune status of TME and predict therapeutic response^13^. For example, TMEscore proposed by Liao *et al.* was reported as an independent prognostic factor and effective in predicting immunotherapeutic outcomes in 1524 GC patients^25^. In addition, the TIDE score was developed to evaluate tumor immune evasion via immune dysfunction and exclusion^26^. Evidence has shown that the TIDE is more accurate to predict response of first-line ICI therapy compared to PD-L1 level or TMB in melanoma patients^26^. However, the two scoring systems did not show a significant predictive power in our study, which may be partially attributed to the relatively small cohort size. Nevertheless, our study observed that *ERBB3* variations correlate with a higher TMEscore and TMEscore A and a lower TMEscore B, which indicate a favorable environment for immunotherapy^12^, further supporting the predictive value of *ERBB3* variations.

We recognize the following limitations in this study that require further investigations. First, the short and diverse follow-up durations among patients may impact the robustness of our findings; therefore, the lack of comprehensive survival analysis limited long-term prognostic assessments. Second, our limited sample and dataset sizes restricted the use of more complex models for feature selection. Future developments in this field could benefit from larger-scale studies with more extensive datasets. These studies could explore the application of more complex feature selection methods, such as LASSO regression and XGBoost, to further refine the prediction model. However, our cautious approach in prioritizing model simplicity and stability aligns with the challenges of our current small dataset. Moreover, the notable differences in clinical settings between the study and validation cohorts, as well as the narrow population focus, restricted the validity and generalizability of our prediction model. Given the advanced nature of our study and challenges associated with data collection in this field, we anticipate future validation studies to be conducted. In addition, predictive markers identified from retrospective studies usually have limited reproducibility in large prospective trials; thus, further clinical investigations are needed. Moreover, bulk transcriptomic data is less representative of intratumoral heterogeneity, which may have compromised predictive power. Further studies using single-cell RNA sequencing would better reflect heterogeneity by characterizing individual cells in tumors. Emerging evidence has shown that antitumor immune response was not only affected by the infiltration levels of immune cells but also their phenotypes. For example, the different levels of PD1 on intratumoral T cells represent distinct phenotypes and are associated with different outcomes of immunotherapy in cancer patients^52^. Therefore, further investigations involving phenotype identifications of the tumor-infiltrating immune cells may enhance the predictive power of these biomarkers. Lastly, considering the evolving landscape of multiomics approaches, future studies might incorporate a broader spectrum of molecular features, such as proteomic profiling for protein signature identification^53^, to enhance the predictive power and broaden the applicability of the prediction model in similar clinical settings.

In conclusion, our study comprehensively evaluated clinicopathological, genetic, and immune features and developed a novel prediction model integrating *CDH1* and *ERBB3* variations, *HLA-DQB1* expression, and naïve CD4+ T cell infiltration. Our findings provide a rational basis for selecting appropriate GC patients with PM for ICI-combined conversion therapy using a prediction model that integrates multigene biomarkers.

## Ethical approval

Ethical approval for this study (IRB-2022-278) was provided by the Ethical Committee of the Zhejiang Cancer Hospital, Hangzhou, China on 11 May 2022.

## Consent

Written informed consent was obtained from the patient for publication of this case report and accompanying images. A copy of the written consent is available for review by the Editor-in-Chief of this journal on request.

## Sources of funding

This study was supported by the Natural Science Foundation of Zhejiang Province of China (LBZ22H160002 awarded to Pengfei Yu).

## Author contribution

P.Y.: conceptualization, funding acquisition, and writing – original draft; G.D. and X.H.: formal analysis, investigation, and writing – original draft; C.W.: formal analysis and writing – review and editing; J.F., L.H., Z.Y., Q.X., X.W., J.Y., Q.O: formal analysis and writing – review and editing; Y.D.: conceptualization, formal analysis, and writing – review and editing; X.C.: conceptualization, supervision, and writing – review and editing.

## Conflicts of interest disclosure

Chenxuan Wang, Xiaoying Wu, Junrong Yan, and Qiuxiang Ou are employees of Nanjing Geneseeq Technology, Inc. All the other authors declared no conflicts of interest.

## Research registration unique identifying number (UIN)

NCT05385809.

## Guarantor

Pengfei Yu and Xiangdong Cheng.

## Data sharing statement

Data generated or analyzed during this study are not publicly available due to ethical reasons but are available from the corresponding author on reasonable request.

## Provenance and peer review

Our paper was not invited.

## Supplementary Material
