# Supplementary material for: Genomic and immune microenvironment features influencing chemoimmunotherapy response in gastric cancer with peritoneal metastasis: a retrospective cohort study
Source: Int J Surg. 2024 Mar 19;110(6):3504–17. doi: 10.1097/JS9.0000000000001281 (PMC11175815; doi:10.1097/JS9.0000000000001281)
Supplement: Supplementary file 2 [file js9-110-3504-s002.docx]

**Supplementary** **Figures**


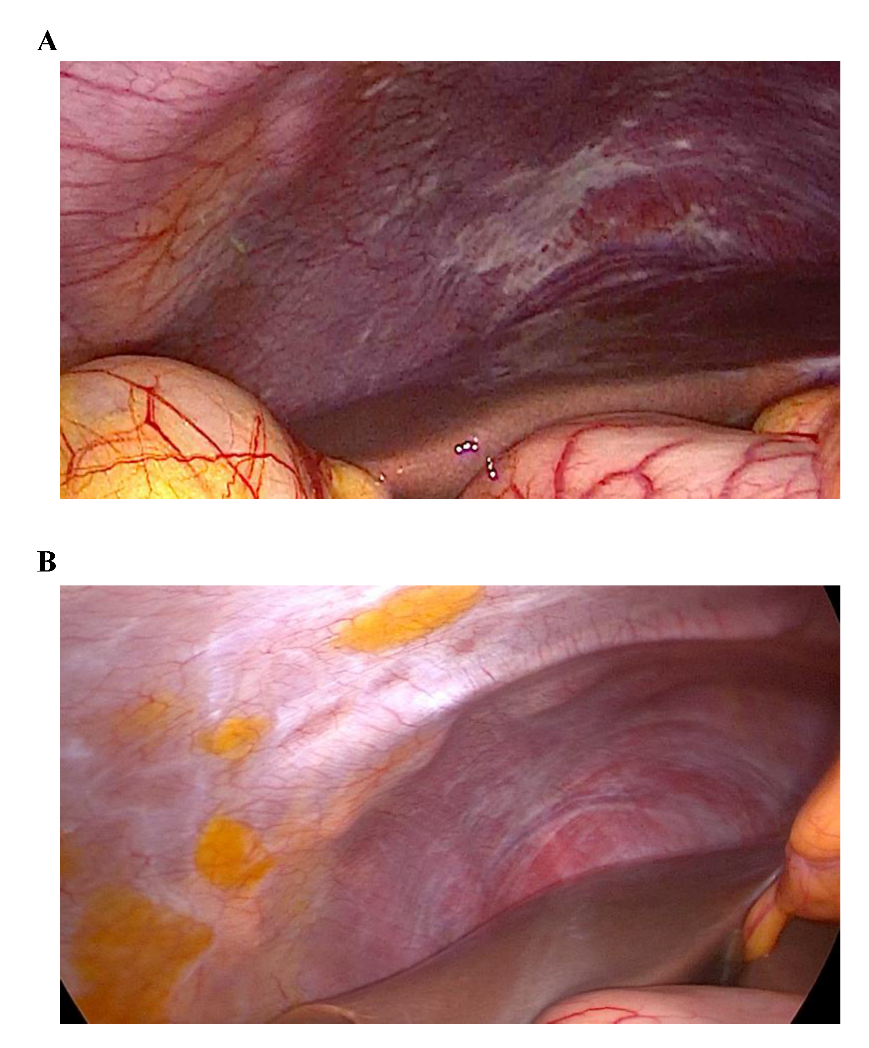


# Supplementary Figure 1.

(**A**) At the first DSL, a flaky metastatic lesion was detected in the right diaphragm, which was confirmed by biopsy as a poorly differentiated adenocarcinoma. (**B**) After two cycles of HIPEC and four cycles of chemoimmunotherapy, the metastasis in the right diaphragm was not obvious, and biopsy suggested scar tissue, with no evidence of tumor.

**
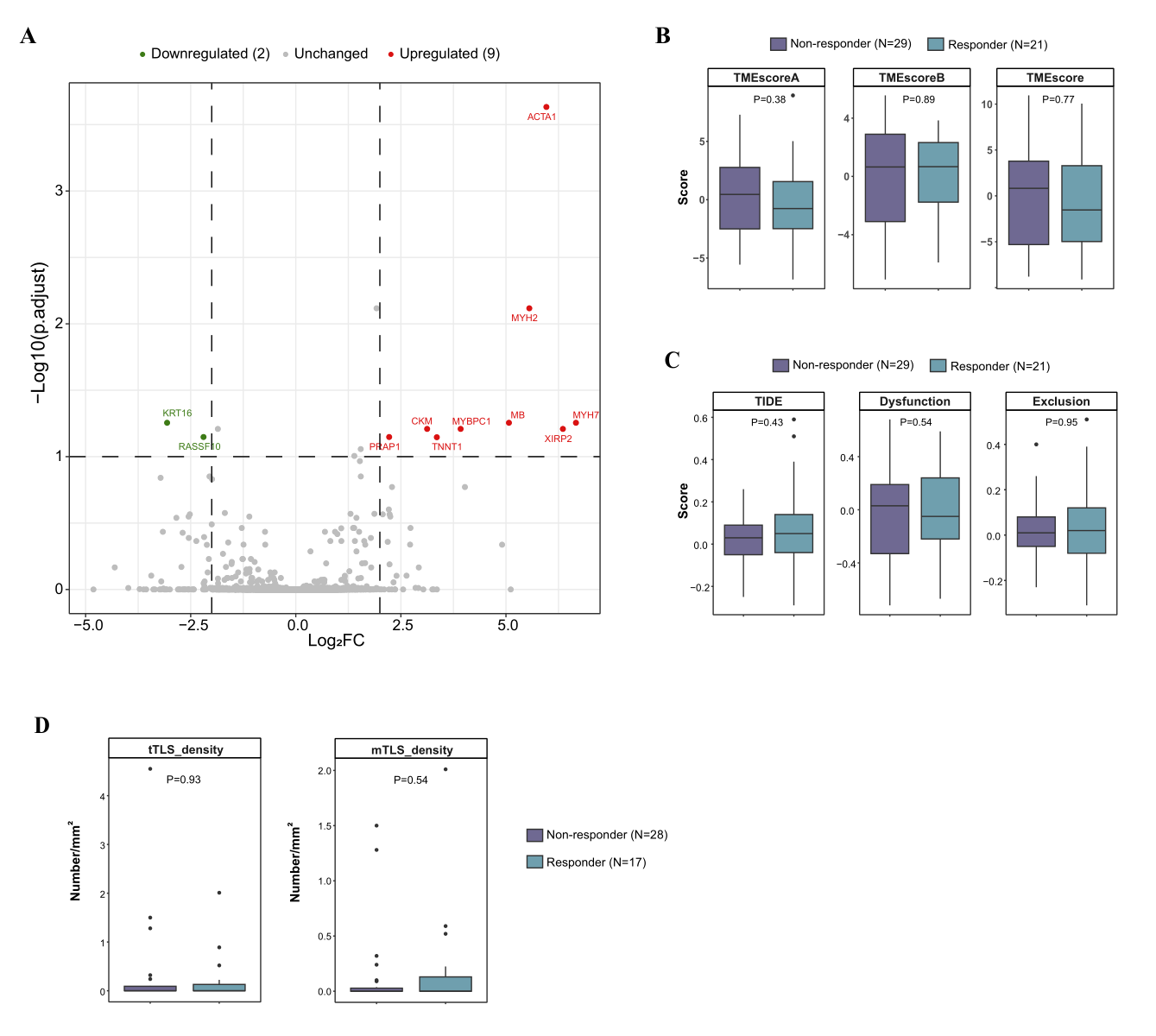
**

# Supplementary Figure 2.

(**A**) RNA-seq analyses identified nine upregulated and two downregulated genes in responders compared to non-responders. The gene expression differences between the two groups were considered significant if |log2FC|≥2 and FDR-adjusted P value (P.adjust)≤0.1. (**B–D**) Group comparisons of TMEscore, TIDE and TLS using a Wilcoxon rank sum test. No significant differences were found. TME: tumor microenvironment; TIDE: Tumor Immune Dysfunction and Exclusion; TLS: tertiary lymphoid structure; t: total; m: mature


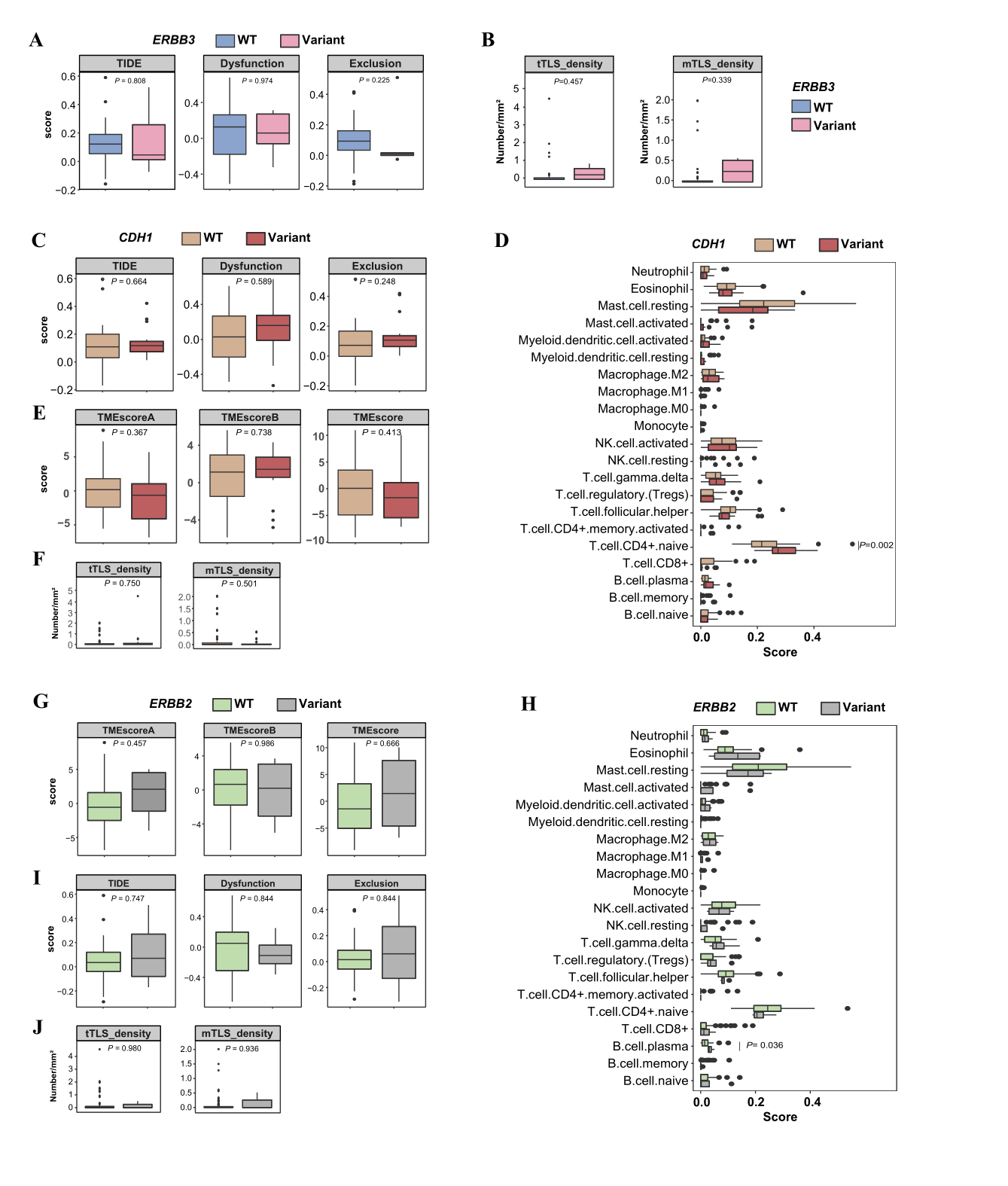


# Supplementary Figure 3.

(**A, B**) Comparisons of TIDE and TLS between patients with ERBB3 variants and wildtype (WT). (**C–F**) Comparisons of TIDE, immune cell infiltration, TMEscore, and TLS between patients with CDH1 variants and WT. (**G–J**) Comparisons of TIDE, immune cell infiltration, TMEscore, and TLS between patients with ERBB2 variants and WT. A Wilcoxon rank sum test was used, and a P-value≤0.05 was considered statistically significant. TME: tumor microenvironment; TIDE: Tumor Immune Dysfunction and Exclusion; TLS: tertiary lymphoid structure; t: total; m: mature


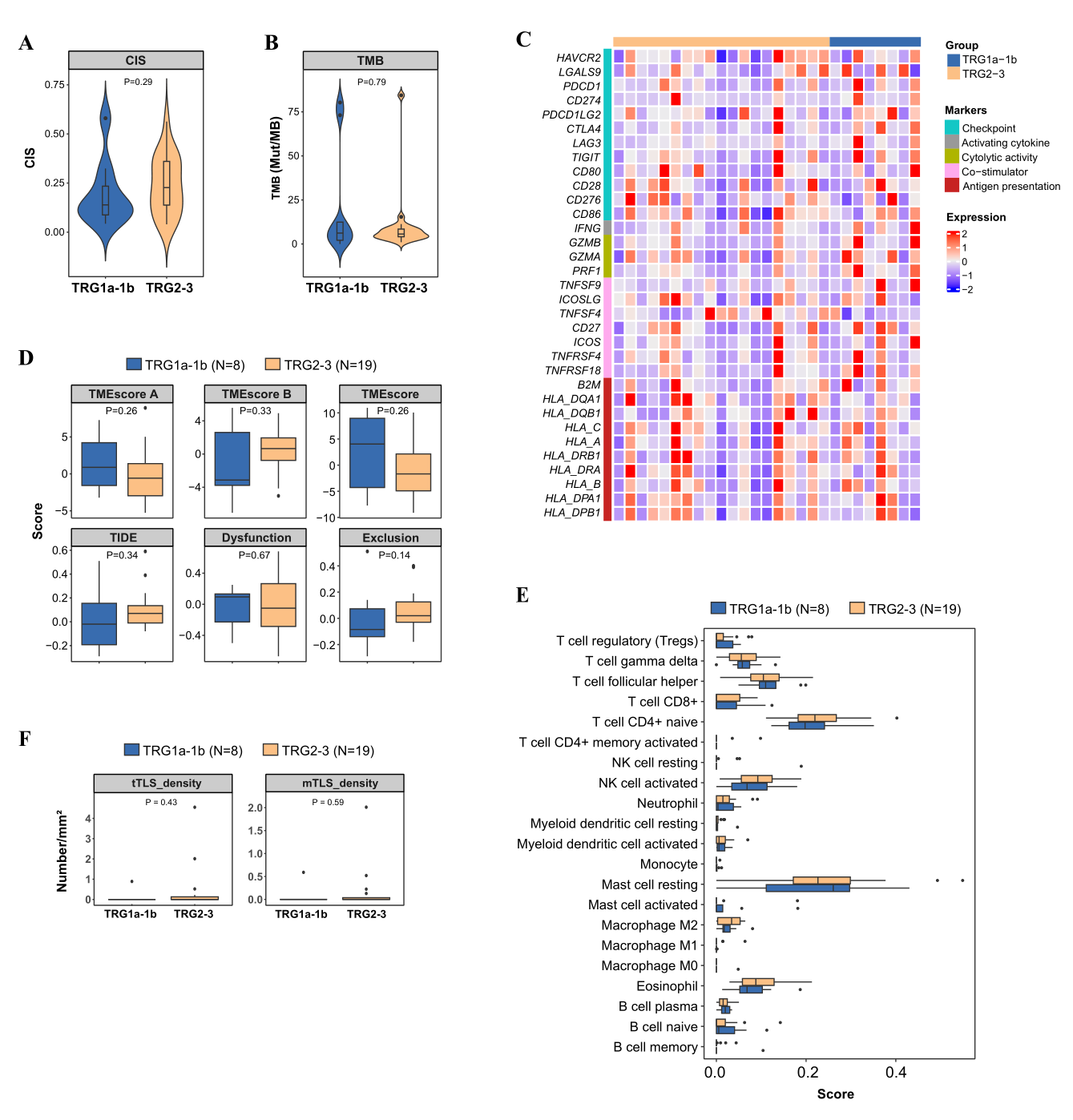


# Supplementary Figure 4.

Comparisons of (**A**) chromosomal instability score (CIS), (**B**) tumor mutational burden (TMB), (**C**) immune-related gene expression, (**D**) TMEscore and TIDE, (**E**) immune cell infiltration, and (**F**) TLS between TRG1a-1b and TRG2-3 group. No significant differences were found. TME: tumor microenvironment; TIDE: Tumor Immune Dysfunction and Exclusion; TLS: tertiary lymphoid structure


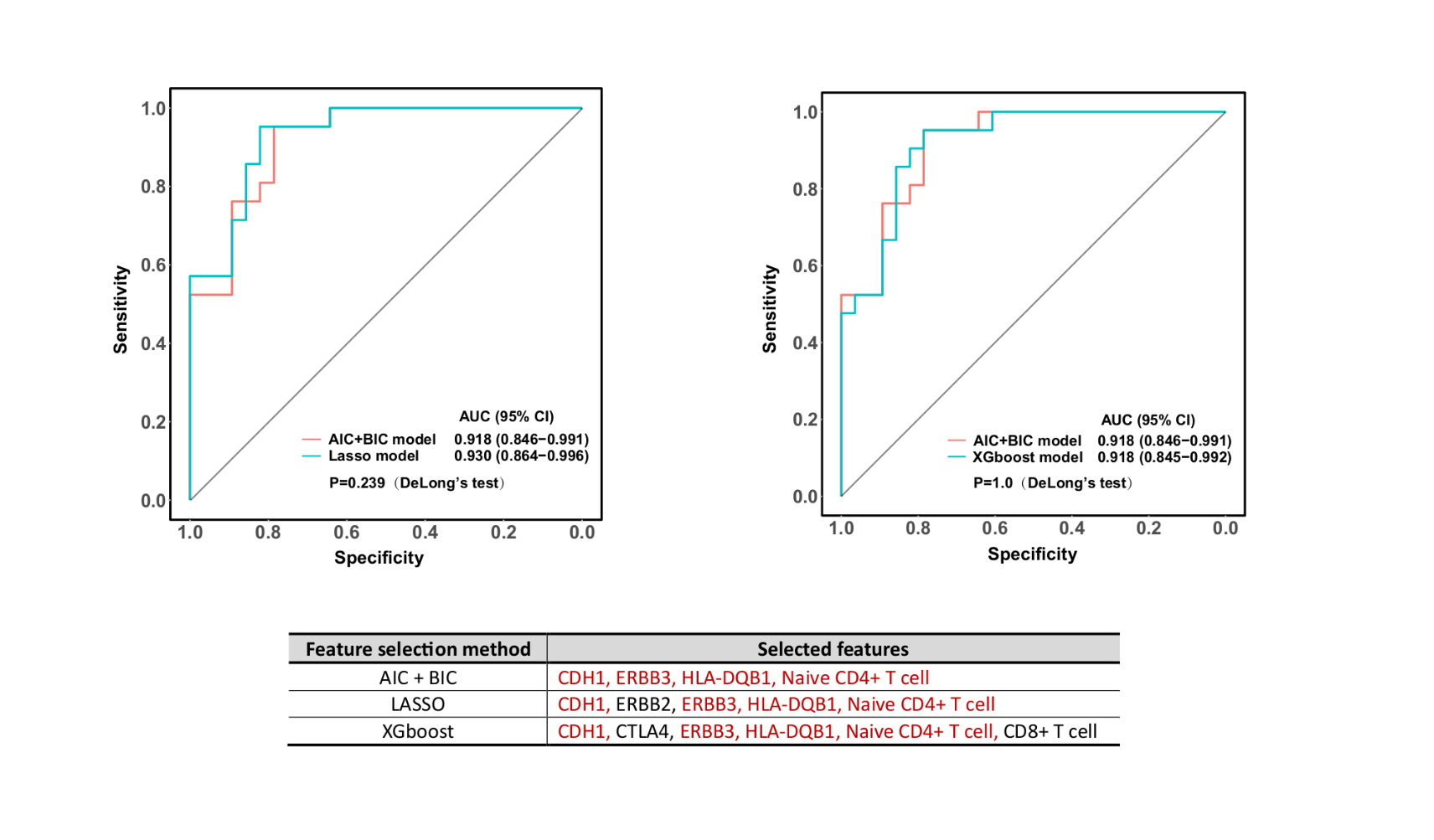


# Supplementary Figure 5.

Model evaluation and comparison based on features selected by AIC+BIC, LASSO, and XGboost. No significant differences were found in AUC between AIC+BIC model and LASSO model. No significant differences were found in AUC between AIC+BIC model and DXBoost model.


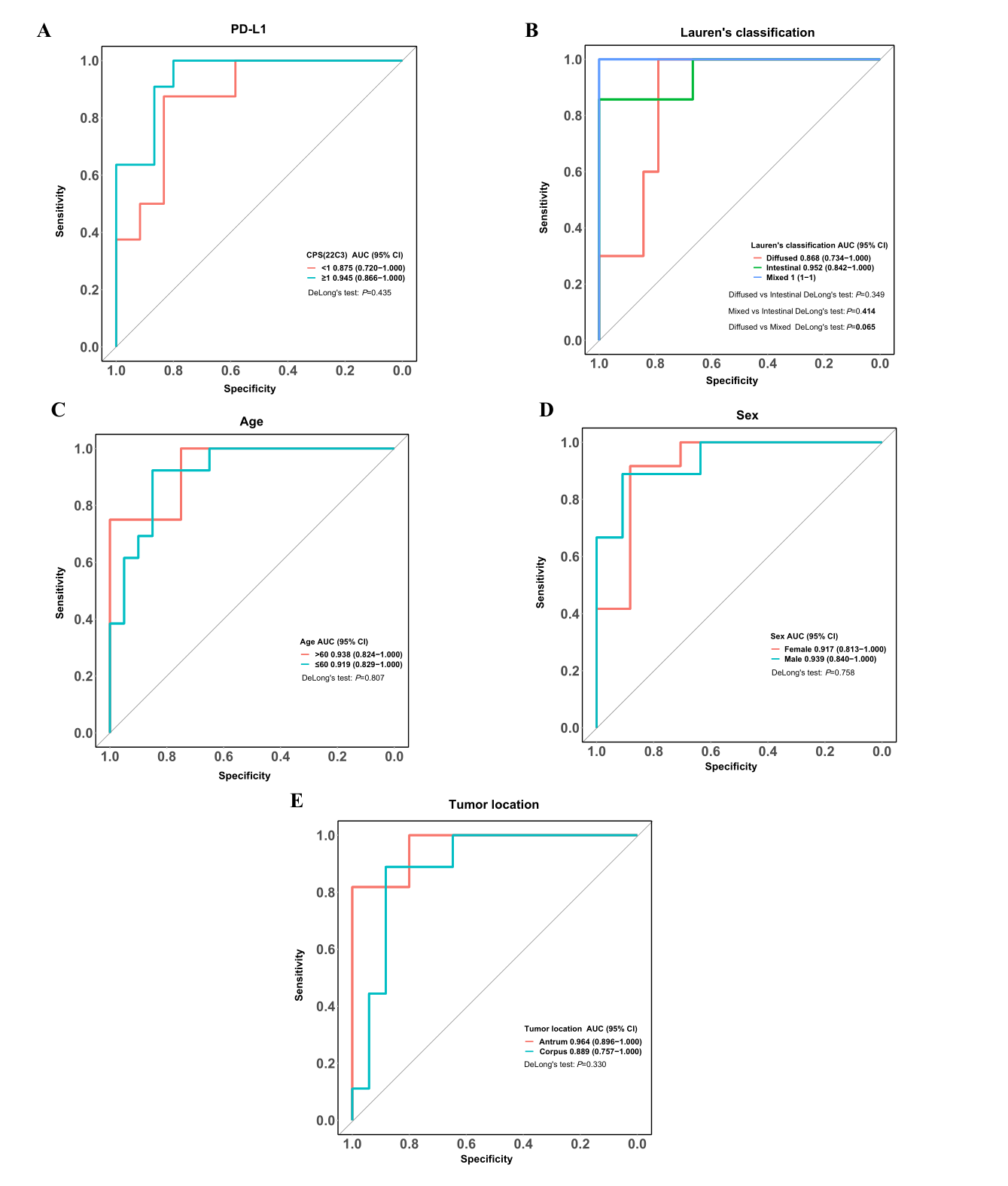


# Supplementary Figure 6.

ROC curves evaluating the performance of the prediction model in patients grouped by clinical characteristics. P values indicate the significance levels of the model’s performance in two matched subgroups analyzed by DeLong’s test.
