# Supplementary material for: Genomic and immune microenvironment features influencing chemoimmunotherapy response in gastric cancer with peritoneal metastasis: a retrospective cohort study
Source: Int J Surg. 2024 Mar 19;110(6):3504–17. doi: 10.1097/JS9.0000000000001281 (PMC11175815; doi:10.1097/JS9.0000000000001281)
Supplement: Supplementary file 3 [file js9-110-3504-s003.docx]

**Supplementary Methods**

## DNA extraction and library preparation

DNA extraction, quantification, and library preparation were performed as previously described.^1^ In brief, DNA was extracted from formalin-fixed paraffin-embedded (FFPE) tumor samples using the QIAamp DNA FFPE Tissue Kit (Qiagen). All DNA samples were qualified using a NanoDrop™2000 spectrophotometer (Thermo Fisher Scientific, Waltham, MA) and quantified using a dsDNA HS Assay Kit (Life Technologies) on a Qubit 3.0 fluorometer. Genomic DNA was sheared into fragments (300–350 bp) using a Covaris M220 instrument. Sequencing libraries were prepared using the KAPA Hyper Prep Kit (KAPA Biosystems). xGen Lockdown Hybridization and Wash Reagents Kit (Integrated DNA Technologies) were used for target enrichment of 437 tumor-related genes using Geneseeq Prime (Nanjing Geneseeq Technology Inc.). The library was captured by Dynabeads M-270 (Life Technologies), amplified in KAPA HiFi HotStart ReadyMix (KAPA Biosystems), and quantified by qPCR using the KAPA Library quantitative Kit (KAPA Biosystems).

## Targeted next generation sequencing (NGS) and data processing

The DNA libraries were paired-end sequenced using Illumina HiSeq4000 NGS platforms (Illumina) as previously described.^1^ Sequencing data were demultiplexed using bcl2fastq (v2.19) and subjected to FASTQ file quality control using Trimmomatic to remove low-quality data and N bases. Subsequently, reads were aligned to the reference human genome hg19 using Burrows-Wheller Aligner. Next, the Genome Analysis ToolKit (GATK) was used for local realignment around indels (insertions/deletions) and base quality score recalibration, followed by deduplication using Picard. The mean sequencing depth was >900× after removing PCR duplicates.

Variant calling and annotations were then performed regarding base substitutions, insertions, deletions, copy number alterations, and structural variation as previously described.^1^ The resulting mutation list was further filtered by an in-house list of recurrent artifacts and common SNPs based on approximately 500 whole blood samples (normal pool) from Chinese cancer patients that were sequenced with the same gene panel at an average depth of 400x. MSI was determined based on the stability of 52 microsatellite sites covered by the targeted NGS panel. TMB was determined by the number of somatic mutations per megabase (Mb) genomic coding area. The chromosomal instability score (CIS) was defined as the proportion of segments with copy number alterations (log_2_ depth ratio>0.2 or <−0.2). Pathway analyses were conducted using the clusterProfiler R package (v4.4.4), and a P ≤ 0.05 was considered significant different between groups. The cancer-related pathways were selected according to a previous study^2^ and KEGG database. The DNA damage response (DDR) pathways were selected as per previously reported.^3^ The immune-related pathways were selected according to ImmPort database.

## RNA sequencing (RNA-seq)

RNA extraction and sequencing were performed as previously described.^4^ In brief, total RNA was extracted from FFPE tumor samples using the RNeasy FFPE Kit (QIAGEN) and quantified using a Bioanalyzer 2100 (Agilent Technologies). Ribosomal RNA and residual genomic DNA were removed using Rnase H and DNase digestion, respectively. Sequencing libraries were prepared using the KAPA Stranded RNA-Seq Library Preparation Kit with RiboErase (HMR) (KAPA Biosystems). Libraries were qualified using the Agilent High Sensitivity DNA kit on a Bioanalyzer 2100 (Agilent Technologies) and subsequently sequenced to a depth of 30 million reads on an Illumina HiSeq NGS platform (Illumina).

## Gene expression and pathway analysis

Basecalling was performed on Illumina bcl2Fastq software (v2.19.0.316) to generate sequence reads in FASTQ format. Sequences were trimmed using Trimmomatic software (v0.36) prior to assembly.^5^ Transcriptomic mapping was performed using STAR software (v2.7.3a) and aligned to the reference human genome (hg19). RSEM (v1.2.31) was used for gene level quantification. Differential expression analysis was performed using the DESeq2 R package based on negative binomial distribution. The gene expression differences between the two groups were considered significant if |log_2_FC|≥2 and FDR-adjusted P value (P.adjust)≤0.1. Gene set enrichment analysis (GSEA), Gene Ontology (GO) term enrichment, and Kyoto Encyclopedia of Genes and Genomes (KEGG) pathway analysis were conducted using the clusterProfiler R package (v4.4.4) with a cut-off of P.adjust<0.05.

## Multiplexed IHC (mIHC) and multispectral imaging

Multiplex immunofluorescence staining was obtained using a PANO 7-plex IHC kit (Panovue, Beijing, China) as per manufacturer’s instructions. Samples were incubated with anti-CD3 (ab135372, Abcam), anti-CD20 (ab78237, Abcam), anti-S100 (GZ031129, Genetech), anti-panCK (CST4545, Cell Signaling Technology), anti- CD21 (ab75985, Abcam), and anti-CD23 (ab92495, Abcam) antibody, followed by horseradish peroxidase-conjugated secondary antibody incubation and tyramide signal amplification. The slides were heat-treated by microwave after each TSA operation. Nuclei were stained with 4′-6′-diamidino-2-phenylindole (DAPI, Sigma-Aldrich). The stained slides were scanned using the Mantra System (PerkinElmer, Waltham, Massachusetts, US), which captures the fluorescent spectra at 20-nm wavelength intervals from 420 to 720 nm with identical exposure time. The scans were combined to build a single stack image. Images of unstained and single-stained sections were used to extract the spectrum of autofluorescence of tissues and each fluorescein, respectively. The extracted images were also used to establish a spectral library for multispectral unmixing using inForm image analysis software (PerkinElmer, Waltham, Massachusetts, US). The spectral library was used to obtain reconstructed images of sections without the autofluorescence.

**References**

1. Yang Z, Yang N, Ou Q, et al. Investigating Novel Resistance Mechanisms to Third-Generation EGFR Tyrosine Kinase Inhibitor Osimertinib in Non-Small Cell Lung Cancer Patients. *Clin Cancer Res*. Jul 1 2018;24(13):3097-3107. doi:10.1158/1078-0432.Ccr-17-2310

2. Sanchez-Vega F, Mina M, Armenia J, et al. Oncogenic Signaling Pathways in The Cancer Genome Atlas. *Cell*. Apr 5 2018;173(2):321-337.e10. doi:10.1016/j.cell.2018.03.035

3. Knijnenburg TA, Wang L, Zimmermann MT, et al. Genomic and Molecular Landscape of DNA Damage Repair Deficiency across The Cancer Genome Atlas. *Cell Rep*. Apr 3 2018;23(1):239-254.e6. doi:10.1016/j.celrep.2018.03.076

4. Dong M, Shan B, Han X, et al. Baseline Mutations and Up-Regulation of PI3K-AKT Pathway Serve as Potential Indicators of Lack of Response to Neoadjuvant Chemotherapy in Stage II/III Breast Cancer. *Front Oncol*. 2021;11:784985. doi:10.3389/fonc.2021.784985

5. Bolger AM, Lohse M, Usadel B. Trimmomatic: a flexible trimmer for Illumina sequence data. *Bioinformatics*. Aug 1 2014;30(15):2114-20. doi:10.1093/bioinformatics/btu170
