# Supplementary material for: Genomic and immune microenvironment features influencing chemoimmunotherapy response in gastric cancer with peritoneal metastasis: a retrospective cohort study
Source: Int J Surg. 2024 Mar 19;110(6):3504–17. doi: 10.1097/JS9.0000000000001281 (PMC11175815; doi:10.1097/JS9.0000000000001281)
Supplement: Supplementary file 4 [file js9-110-3504-s004.docx]

**Supplementary** **Tables**

# Supplementary Table 1. Patient characteristics

| **Characteristics** | **Number of patients (%)** |
| --- | --- |
| **All patients** | N=55 |
| **Age of diagnosis** | |
| ≤60 | 37 (67.3%) |
| >60 | 18 (32.7%) |
| Median (range, years) | 55 (26-75) |
| **Sex** | |
| Female | 30 (54.5%) |
| Male | 25 (45.5%) |
| **Smoking history** | |
| Without | 46 (83.6%) |
| With | 9 (16.4%) |
| **Drinking history** | |
| Without | 48 (87.3%) |
| With | 7 (12.7%) |
| **Tumor site** | |
| Antrum | 22 (40.0%) |
| Cardia | 1 (1.8%) |
| Corpus | 30 (54.5%) |
| Fundus | 2 (3.6%) |
| **Histological grade** | |
| Poorly differentiated | 44 (80.0%) |
| Medium-low differentiated | 8 (14.5%) |
| Moderately differentiated | 3 (5.5%) |
| **Lauren's classification** | |
| Diffused | 31 (56.4%) |
| Intestinal | 14 (25.4%) |
| Mixed | 10 (18.2%) |
| **CA199 (U/mL)** | |
| ≤37 | 39 (70.9%) |
| >37 | 16 (29.1%) |
| **CEA (ng/mL)** | |
| ≤5 | 45 (81.8%) |
| >5 | 10 (18.2%) |
| **CA125 (U/mL)** | |
| ≤35 | 20 (36.4%) |
| >35 | 35 (63.6%) |
| ***ERBB2* amplification (FISH/IHC)** | |
| No | 39 (95.1%) |
| Yes | 2 (4.9%) |
| Unknown | 14 |
| **MMR status (IHC)** | |
| pMMR | 39 (92.9%) |
| dMMR | 3 (7.1%) |
| Unknown | 13 |
| **PD-L1 (22C3, CPS)** | |
| <1 | 21 (42.0%) |
| ≥1 | 29 (58.0%) |
| Unknown | 5 |

CEA: carcinoembryonic antigen; CA: cancer antigen; pMMR: proficient mismatch repair; dMMR: deficient mismatch repair; CPS: combined positive score.

# Supplementary Table 2. Chemoimmunotherapy-related adverse events

|  | **Number of patients (%)** | | |
| --- | --- | --- | --- |
| **Adverse events** | **Grade 1–2** | **Grade 3–4** | **Total** |
| **Overall** | 30 (54.5%) | 16 (29.1%) | 46 (83.6%) |
| **Leucopenia/neutropenia** | 5 (9.1%) | 9 (16.4%) | 14 (25.5%) |
| **Anemia** | 13 (23.6%) | 2 (3.6%) | 15 (27.3%) |
| **Thrombocytopenia** | 4 (7.3%) | 2 (3.6%) | 6 (10.9%) |
| **Elevated ALT/AST** | 2 (3.6%) | 5 (9.1%) | 7 (12.7%) |
| **Nausea/vomiting** | 8 (14.5%) | 3 (5.5%) | 11 (20%) |
| **Pneumonia/pneumonitis** | 3 (5.5%) | 0 | 3 (5.5%) |
| **Rash** | 5 (9.1%) | 0 | 5 (9.1%) |
| **Fatigue** | 10 (18.2%) | 0 | 10 (18.2%) |
| **Elevated creatinine** | 3 (5.5%) | 0 | 3 (5.5%) |
| **Hypothyroidism** | 7 (12.7%) | 0 | 7 (12.7%) |

ALT: alanine aminotransferase, AST: aspartate aminotransferase
